# The HERC2 ubiquitin ligase is essential for embryonic development and regulates motor coordination

**DOI:** 10.18632/oncotarget.11270

**Published:** 2016-08-12

**Authors:** Monica Cubillos-Rojas, Taiane Schneider, Ouadah Hadjebi, Leonardo Pedrazza, Jarbas Rodrigues de Oliveira, Francina Langa, Jean-Louis Guénet, Joan Duran, Josep Maria de Anta, Soledad Alcántara, Rocio Ruiz, Eva María Pérez-Villegas, Francisco J. Aguilar, Ángel M. Carrión, Jose Angel Armengol, Emma Baple, Andrew H. Crosby, Ramon Bartrons, Francesc Ventura, Jose Luis Rosa

**Affiliations:** ^1^ Departament de Ciències Fisiològiques, IDIBELL, Campus de Bellvitge, Universitat de Barcelona, L'Hospitalet de Llobregat, Barcelona, Spain; ^2^ Laboratório de Pesquisa em Biofísica Celular e Inflamação, Pontifícia Universidade Católica do Rio Grande do Sul, Porto Alegre, Rio Grande do Sul, Brazil; ^3^ Département de Biologie du Développement, Institut Pasteur, Paris, France; ^4^ Departament de Patologia i Terapèutica Experimental, Campus de Bellvitge, Universitat de Barcelona, L'Hospitalet de Llobregat, Barcelona, Spain; ^5^ Departamento de Bioquímica y Biología Molecular, Facultad de Farmacia, Universidad de Sevilla, Sevilla, Spain; ^6^ Departamento de Fisiología, Anatomía y Biología Celular, Universidad Pablo de Olavide, Sevilla, Spain; ^7^ Institute of Biomedical and Clinical Science, University of Exeter Medical School, RILD Wellcome Wolfson Centre, Exeter, UK

**Keywords:** ubiquitin, p53, Angelman syndrome, Purkinje cells, behavioural analysis, Pathology Section

## Abstract

A mutation in the *HERC2* gene has been linked to a severe neurodevelopmental disorder with similarities to the Angelman syndrome. This gene codifies a protein with ubiquitin ligase activity that regulates the activity of tumor protein p53 and is involved in important cellular processes such as DNA repair, cell cycle, cancer, and iron metabolism. Despite the critical role of HERC2 in these physiological and pathological processes, little is known about its relevance *in vivo*. Here, we described a mouse with targeted inactivation of the *Herc2* gene. Homozygous mice were not viable. Distinct from other ubiquitin ligases that interact with p53, such as MDM2 or MDM4, p53 depletion did not rescue the lethality of homozygous mice. The HERC2 protein levels were reduced by approximately one-half in heterozygous mice. Consequently, HERC2 activities, including ubiquitin ligase and stimulation of p53 activity, were lower in heterozygous mice. A decrease in HERC2 activities was also observed in human skin fibroblasts from individuals with an Angelman-like syndrome that express an unstable mutant protein of HERC2. Behavioural analysis of heterozygous mice identified an impaired motor synchronization with normal neuromuscular function. This effect was not observed in p53 knockout mice, indicating that a mechanism independent of p53 activity is involved. Morphological analysis showed the presence of HERC2 in Purkinje cells and a specific loss of these neurons in the cerebella of heterozygous mice. In these animals, an increase of autophagosomes and lysosomes was observed. Our findings establish a crucial role of HERC2 in embryonic development and motor coordination.

## INTRODUCTION

Angelman syndrome (AS) is a severe neurodevelopmental disorder that occurs in approximately one out of every 12,000 births. Patients with AS exhibit developmental delay, speech impairments, intellectual disability, epilepsy, abnormal electroencephalograms, puppet-like ataxic movements, prognathism, tongue protrusion, paroxysms of laughter, abnormal sleep patterns, hyperactivity, and a high prevalence of autism [1, 2]. Genetic studies revealed that AS is associated with maternal deletions of chromosome 15q11-q13, paternal chromosome 15 uniparental disomy, or rare imprinting defects that affect the transcription of genes within the 15q11-q13 region. Specific loss-of-function mutations in the maternally inherited *UBE3A* gene which resides within this chromosomal region have been identified in a subset of affected individuals [3]. The *UBE3A* gene encodes an E3 ubiquitin ligase called UBE3A or E6-associated protein (E6AP). More recently, a mutation in the *HERC2* gene has been linked to neurodevelopmental delay and dysfunction in both AS and autism-spectrum disorders among the Old Order Amish [4, 5]. Molecular analysis associated a missense mutation in the *HERC2* gene (c.1781C>T, p.Pro594Leu) with the disease phenotype. Although the *HERC2* gene also resides in the 15q11-q13 region, it seems that it is not imprinted [6]. *HERC2* encodes an ubiquitin ligase that binds to UBE3A and stimulates its ubiquitin ligase activity [7]. Deregulation of the activity of UBE3A is well recognized as contributing to the development of AS [2, 3]. Thus, disruption of HERC2 function by this mutation is associated with a reduction in UBE3A activity resulting in neurodevelopmental delay with Angelman-like features [4, 5].

Genetic variations in the *HERC2* gene are associated with eye pigmentation. Although multiple genes contribute to eye colour in humans, most variation can be attributed to a strong interaction between *HERC2* and adjacent *OCA2* on chromosome 15 [8]. A distal regulatory element of the *OCA2* promoter is within intron 86 of the *HERC2* gene and three different sequence variants of *HERC2* have been identified, such as predictors of eye colour in humans [9, 10].

*HERC2* belongs to the *HERC* gene family that encodes a group of proteins that contain multiple structural domains. All members have at least one copy of an N-terminal region showing homology to the cell cycle regulator RCC1 and a C-terminal HECT (homologous to the E6-AP carboxyl terminus) domain found in a number of E3 ubiquitin protein ligases. These two domains define the HERC family (HERC = HECT + RCC1) [11]. In humans, six members form the HERC family. They are classified into two groups: large (HERC1-2) and small (HERC3-6) proteins. Structurally, small HERC proteins contain the two characteristic domains HECT and RCC1, whereas large HERC proteins are giant proteins (approximately 5,000 amino acid residues) containing additional domains, including several RCC1 domains. Functionally, the HERC protein family regulates ubiquitination and ISGylation processes associated with membrane trafficking, immune response, DNA repair, cell stress response and cancer biology [11–20]. Recently, several substrates of HERC2 have been identified. HERC2 targets ubiquitin-dependent proteasomal degradation to xeroderma pigmentosa A (XPA) during circadian control of nucleotide excision repair [21] and the breast cancer suppressor BRCA1 during the cell cycle [22]. These data, together with the interaction of HERC2 with RNF8 [23], indicate a regulatory role for HERC2 in DNA repair by nucleotide excision and by homologous recombination of DNA double-strand breakage. More recently, other substrates, such as NEURL4, USP33 or FBXL5, have been reported that also indicate the participation of HERC2 in other important cellular processes such as centrosome architecture, β-adrenergic receptor recycling, RalB signaling, cancer cell migration, and iron metabolism [24–26].

HERC2 may also interact with proteins in a manner independent of proteasomal degradation. The tumor suppressor p53 is a transcription factor that coordinates the cellular response to several kinds of stress through the regulation of a wide range of genes [27, 28]. In response to stress, p53 transcriptional activation is dependent of its oligomerization state [29]. Thus, p53 mutations that impair its oligomerization have been associated with a rare hereditary cancer predisposition disorder called Li-Fraumeni syndrome [30, 31]. HERC2 interacts with p53 and modulates its transcriptional activity by regulating its oligomerization [32]. RNA interference experiments showed that HERC2 knock-down inhibited p53 oligomerization affecting its transcriptional activity. Under these conditions, up-regulation of cell growth and increased focus formation were observed, suggesting an important role of HERC2 in proliferation [32]. In agreement with these observations, an association of frameshift mutations of *HERC2* with gastric and colorectal carcinoma has been described [18]. Despite the critical role of HERC2 in cellular processes regulated by p53, little is known about its physiological relevance. The mutation of HERC2 found among the Old Order Amish with features similar to Angelman syndrome also suggests an important role for HERC2 in neurodevelopment [4, 5]. To determine the physiological importance of HERC2, we decided to generate a mouse with targeted inactivation of the *Herc2* gene.

## RESULTS

### Characterization of the *Herc2*^530^ mice

**Figure 1:**
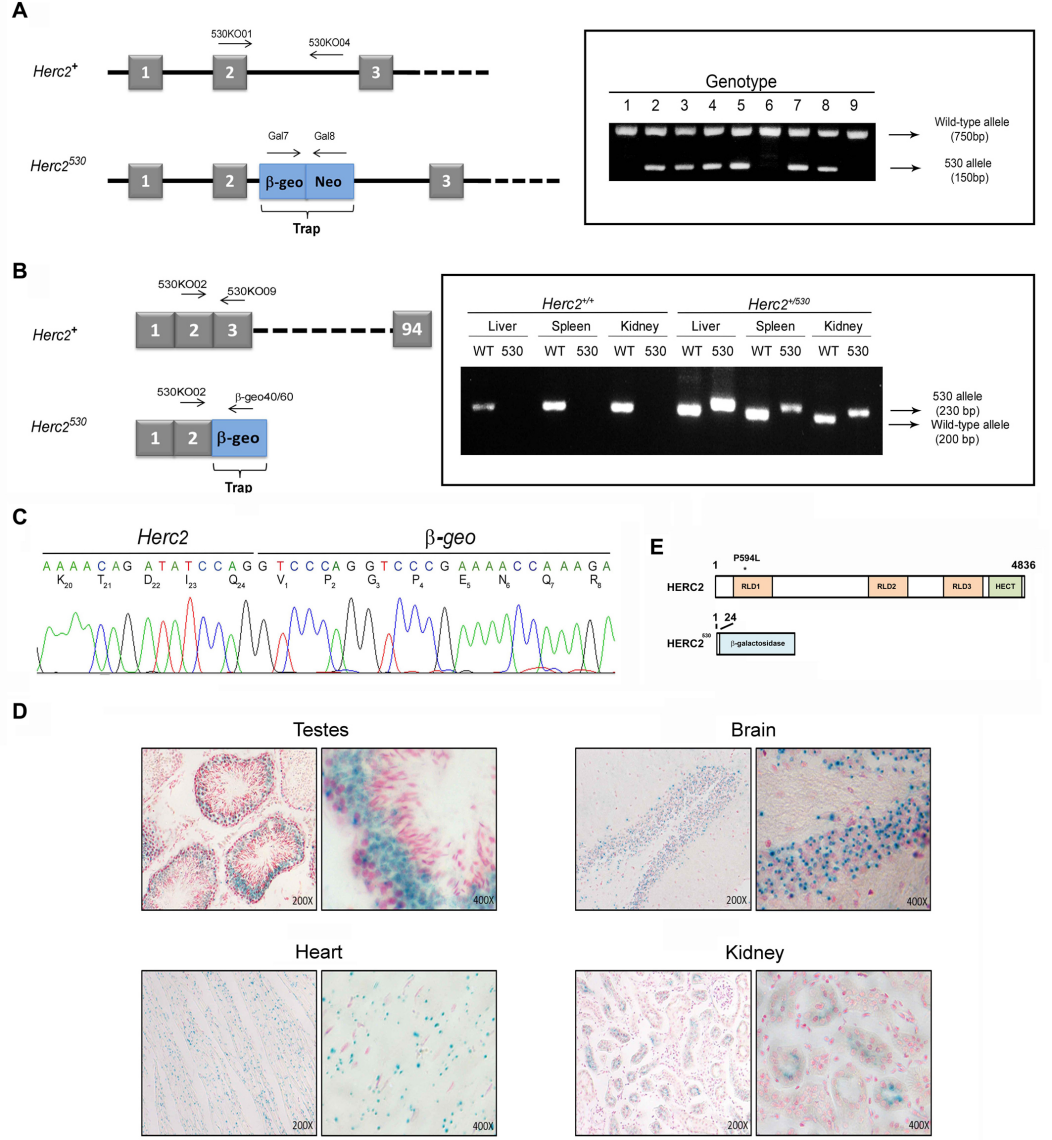
Generation of the *Herc2*^**530**^ mice **A.** Schematic representation of the *Herc2* wild-type allele (*Herc2^+^*) and *Herc2^530^* allele and the designed primers to identify both alleles (left). The *Herc2^530^* allele contains the pGT0lxr vector that expresses the fusion of β-galactosidase and neomycin transferase within intron 2. The integration of the trap was determined by genotyping using the indicated primers (right), 530KO01/530KO04 for the wild-type allele and Gal7/Gal8 for the 530 allele. **B.** Exon structure of *Herc2^+^* and *Herc2^530^* (left). RT-PCR experiments with mRNA from liver, spleen and kidney of *Herc2^+^* and *Herc2^530^* mice was performed using the indicated primers. **C.** PCR products from B were sequenced. The trap was inserted after exon 2, for which the mutant protein contains the first 24 amino acids of the HERC2 and β-galactosidase protein. **D.** β-galactosidase expression in *Herc2^530^* mice. The activity of β-galactosidase was determined in the testes, brain, heart and kidney of *Herc2^530^* mice and detected by *X-gal* staining. **E.** Scheme of HERC2 protein and the expected product from the *Herc2^530^* allele. The P594L pathological mutation is indicated (*). RLD: RCC1-like domain.

To study the physiological role of HERC2, we generated a novel mutant allele at the *Herc2* locus by using a gene trapped embryonic stem (ES) cell line from The Sanger Institute. These ES cells, here called *Herc2*^530^, contain a pGT0lxr expression vector with a strong splice acceptor site integrated within intron 2 of the mouse *Herc2* gene, which results in the expression of a truncated mRNA. PCR experiments with genomic DNA from the tails of mice generated with these ES cells confirmed the integration of the trap between exon 2 and 3 (Figure 1A). RT-PCR experiments with mRNA from different tissues showed the formation of a truncated mRNA of *Herc2* fused to β*-galactosidase* (β-geo) (Figure 1B). Sequencing analysis confirmed these results and revealed the fusion of β-galactosidase after amino acid residue 24 of HERC2 (Figure 1C). β-galactosidase activity was determined in several tissues (Figure 1D), confirming the expression of a fused transcript of the first 24 amino acid residues of HERC2 with β-galactosidase. Because mouse HERC2 protein has 4,836 amino acid residues, we can consider that the HERC2 new mutant allele *Herc2*^530^ is functionally deleted (Figure 1E).

### *Herc2* is an essential gene during embryonic development

**Figure 2:**
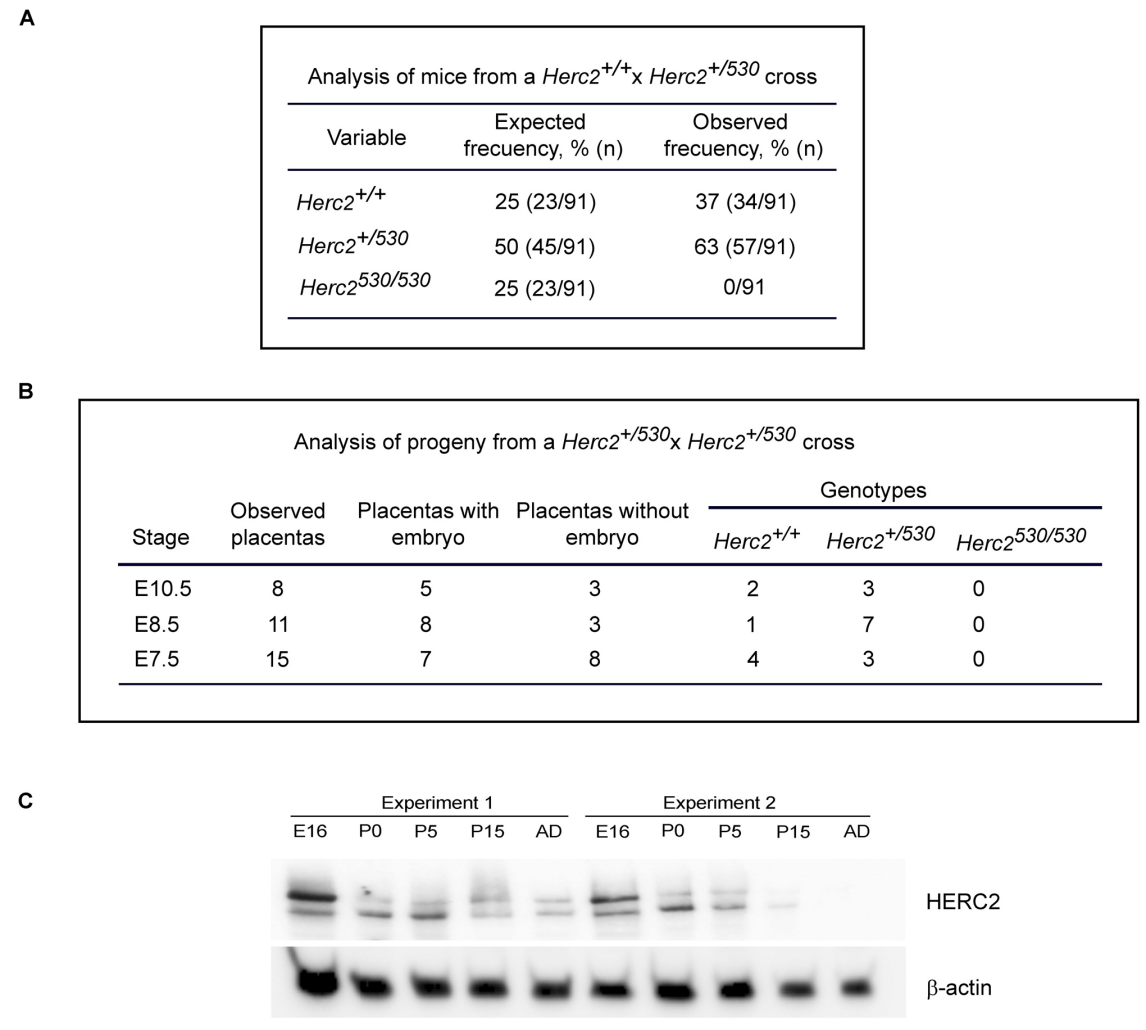
Analysis of progeny from the *Herc2^+/530^* cross **A.** Analysis of offspring born from the intercross of *Herc2^+/530^* mice. Ninety-one animals were genotyped by PCR of genomic DNA isolated from mouse tails. The expected frequencies for *Herc2^+/+^* and *Herc2^+/530^* were obtained; however, no homozygous mice (*Herc2^530/530^*) were identified. **B.** Analysis of embryonic lethality in *Herc2^530/530^* mice. Embryos from *Herc2^+/530^* pregnant females at different stages were isolated and genotyped. The *Herc2^+/+^* and *Herc2^+/530^* genotypes were identified, but not the *Herc2^530/530^*. The placentas without embryos could not be genotyped. **C.** Analysis of HERC2 protein levels during development. Lysates from brains at different stages were analyzed by immunoblotting for HERC2 and β-actin. E16 (embryonic day), P0, P5 and P15 (post-natal day 0, 5 and 15, respectively) and AD (adult animal).

91 offspring born from the intercross heterozygous mice were genotyped by PCR. Among these mice, 34 (37%) were wild-type for *Herc2*, and 57 (63%) were heterozygous for *Herc2^+/530^* (Figure 2A). We could not identify any viable homozygous *Herc2^530/530^*. These data suggested an embryonic lethality for null mice. To determine the time of embryonic lethality in *Herc2^530/530^* mice, genomic DNA was isolated from embryos harvested at different stages (E7.5, E8.5 and E10.5) of pregnancy from *Herc2^+/530^* mice. We isolated 34 placentas and observed 14 (41%) without embryos (Figure 2B). Among the embryos, we identified both wild-type and heterozygous mice, but not homozygous mice. At day 7.5 and 8.5 in placentas without embryos, we observed some residuals. At day 10.5, these residuals were completely resorbed. Most likely, these residuals were rests of embryos homozygous for *Herc2^530^*. These results indicate that the expression of at least a normal *Herc2* copy is essential for the completion of embryonic development before day 7.5.

Genes important for development are usually highly expressed during embryonic stages. To determine the expression pattern of the HERC2 protein during development, we analyzed the levels of the endogenous protein in samples from brains at embryonic day E16, postnatal days P1, P5 and P15, and adult mice (8 weeks). Anti-HERC2 antibodies detected a double band that decreased during development, with the highest levels in the embryonic stage and lower levels in the adult animal (Figure 2C). Similar expression profiles were observed for other members of the HERC family, such as HERC1 or HERC3 (data not shown), suggesting an important role of the HERC proteins during embryonic development.

### *p53* inactivation did not rescue embryonic lethality of homozygous *Herc2^530^* mice

**Figure 3:**
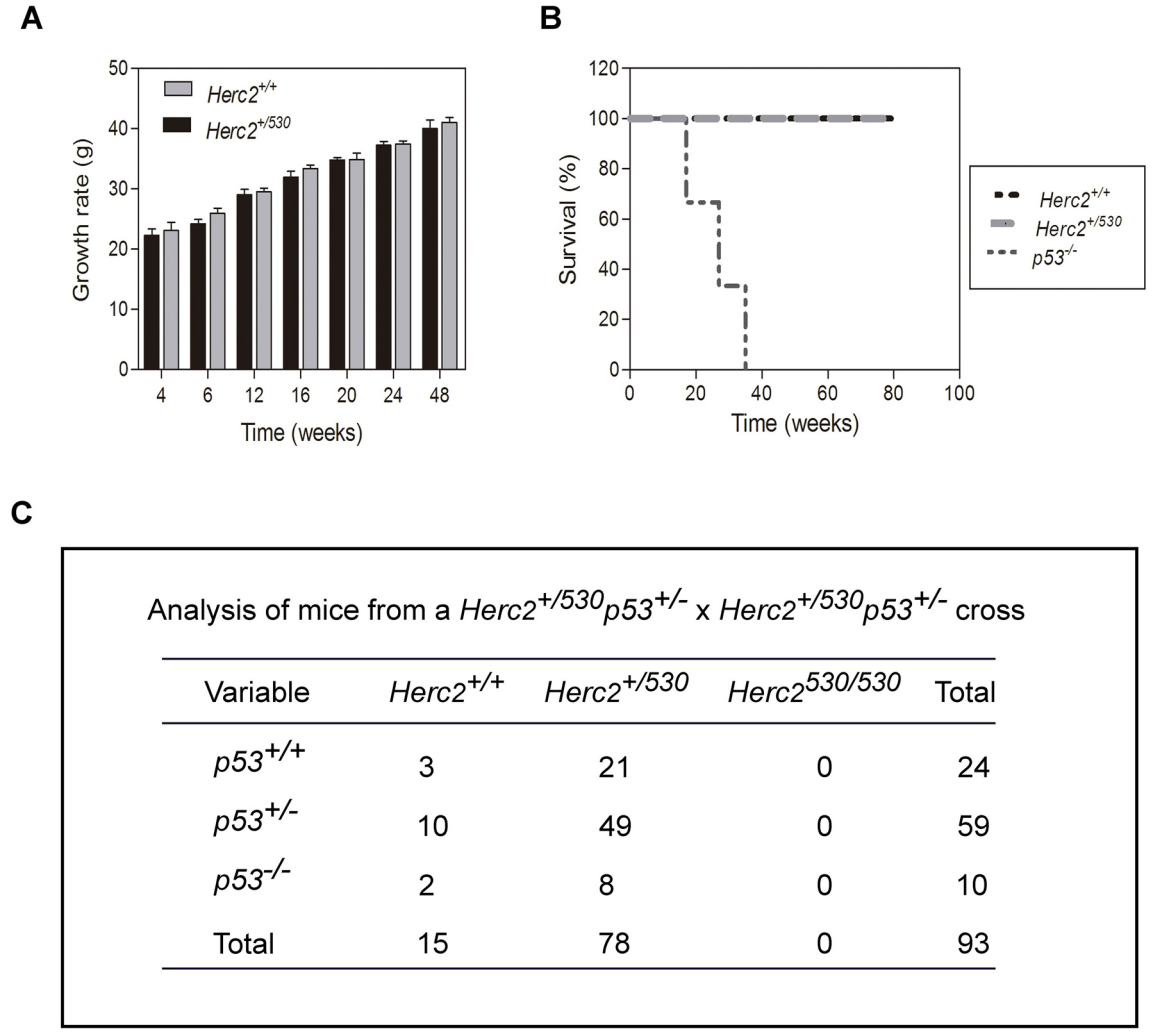
*p53* inactivation did not rescue the lethality of *Herc2^530/530^* homozygous mice Graphs of growth rate **A.** and survival **B.** from *Herc2^+/+^* and *Herc2^+/530^* mice. Growth and survival were analyzed in male mice (n>10) at the indicated weeks. The survival for *p53^−/−^* mice also was analyzed. **C.** The analysis of mice from a cross of double heterozygous *Herc2^+/530^ p53^+/−^* animals. The offspring was genotyped by PCR of genomic DNA with the appropriate primers, indicating that the embryonic lethal phenotype of *Herc2^530/530^* embryos was not rescued by crossing with *p53^−/−^* mice.

Growth curves and survival rates were analyzed in *Herc2^+/530^* mice. The growth curve was not significantly altered in *Herc2^+/530^* mice during the time studied (Figure 3A). The survival rate was also similar to wild-type mice during the period studied (Figure 3B).

It had been previously demonstrated that knockout mice for the E3 ubiquitin ligases of p53, such as *MDM2* or *MDM4*, are lethal in embryonic stages due to growth inhibition and apoptosis. Interestingly, this lethality could be rescued by concomitant p53 depletion [33–35]. Because HERC2 is an E3 ubiquitin ligase that regulates p53 activity [32], we asked whether p53 inactivation might also rescue the lethality of *Herc2^530/530^* homozygous mice. We crossed double heterozygous *Herc2* and *p53* mice (*Herc2*^+^*^/530^ p53^+/−^* mice) to obtain double homozygous mice. We analyzed the genotype of the offspring by performing an adequate PCR assay on of genomic DNA samples but did not observe any viable *Herc2^530/530^* mouse (Figure 3C). These data suggest the non-involvement of p53 in the embryonic death caused by the depletion of *Herc2*.

### Partial inactivation of the *Herc2* gene is sufficient to reduce HERC2 protein levels and activity

**Figure 4:**
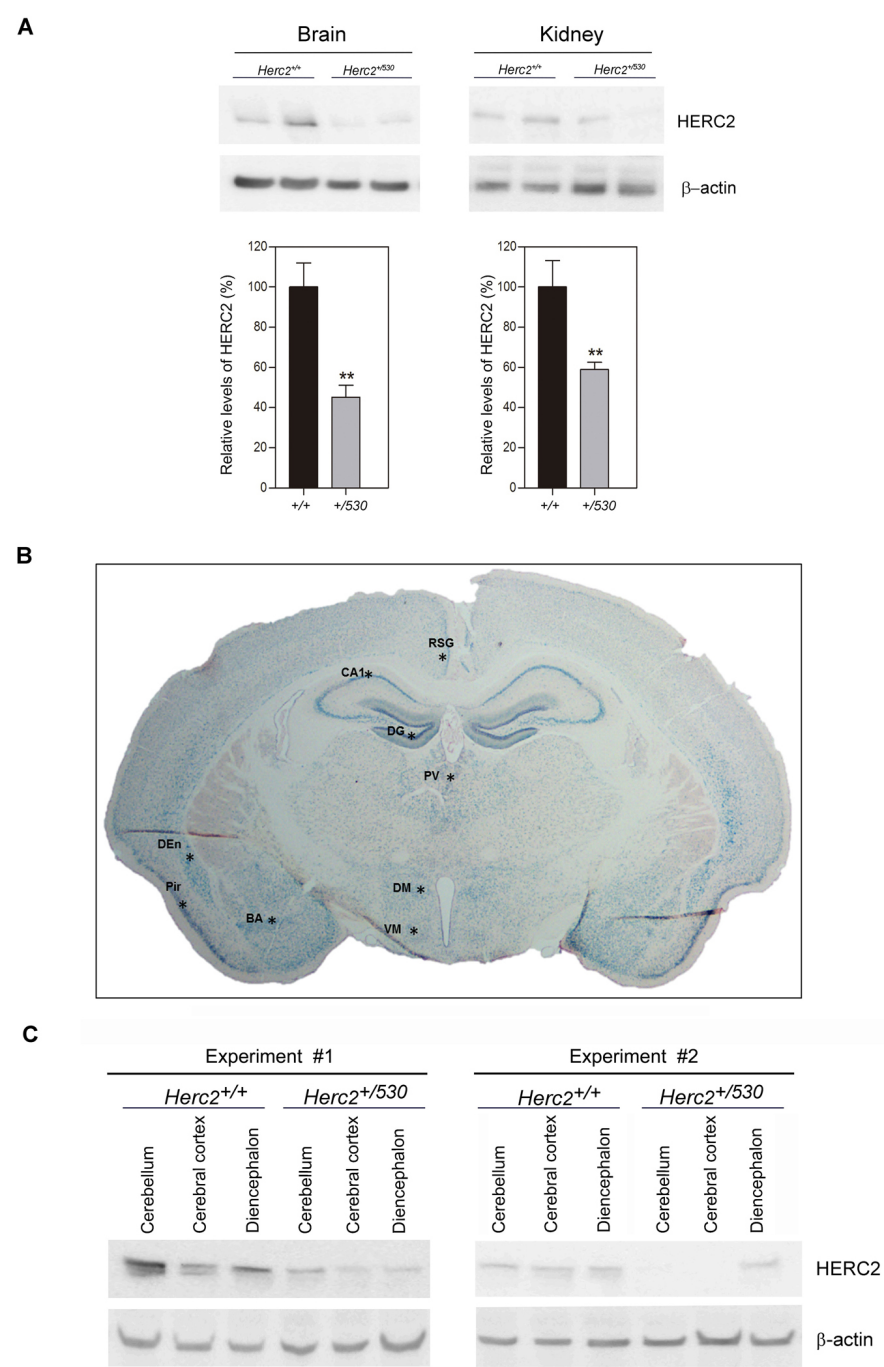
*Herc2^+/530^* mice show reduced levels of HERC2 protein **A.** HERC2 protein levels were analyzed by immunoblotting using specific antibodies against HERC2 in several tissues from 8 week old mice. The levels of HERC2 were quantified (*n* = 8) and normalized with respect to β-actin levels. **B.** β-galactosidase expression in brain from *Herc2^530^* mouse. The β-galactosidase activity was detected ubiquitously in all areas using *X-gal* staining. However, there were forebrain cortical and subcortical areas in which β-galactosidase labeling was the most intense (asterisks). **C.** The levels of HERC2 were analyzed by immunoblotting in lysates of cerebellum, cerebral cortex and diencephalon from *Herc2^+/+^* and *Herc2^+/530^* mice at P4 (post-natal day 4). BA, basal amygdala. CA1, pyramidal cell layer of the hippocampal cornu ammonis 1. DEn, dorsal endopiriform nucleus. DG, granular cell layer of hippocampal dentate gyrus. DM, dorsomedial nucleus of the hypothalamus. Pir, piriform cortex. PV, paraventricular thalamic nucleus. RSG, retrosplenial granular cortex. VM, ventromedial nucleus of the hypothalamus.

We analyzed protein levels of HERC2 in *Herc2^+/530^* animals by immunoblotting. We observed that the levels of HERC2 were decreased approximately 50% in the brain and kidney of *Herc2^+/530^* animals (Figure 4A). The analysis of β-galactosidase activity in *Herc2^+/530^* mice allows the analysis of *Herc2* expression in more detail. Ubiquitous expression of HERC2 was observed in all tissues analyzed (Figure 1D). In the brain, this expression was higher in the hippocampus (pyramidal cell layer and granular layer of dentate gyrus), hypothalamic nucleus (dorsomedial and ventromedial), amygdaloid nucleus (basal and medial), piriform cortex, dorsal endopiriform nucleus, entorhinal cortex, retrospenial cortex, paraventricular thalamic nucleus, and cerebellum (Figure 4B and data not shown). These observations were confirmed by immunoblotting. We dissected the cerebellum, cerebral cortex and diencephalon, detecting HERC2 protein in all these areas (Figure 4C). The levels of HERC2 protein were reduced in *Herc2^+/530^* animals (Figure 4C) with respect to control mice (*Herc2^+/+^*).

**Figure 5:**
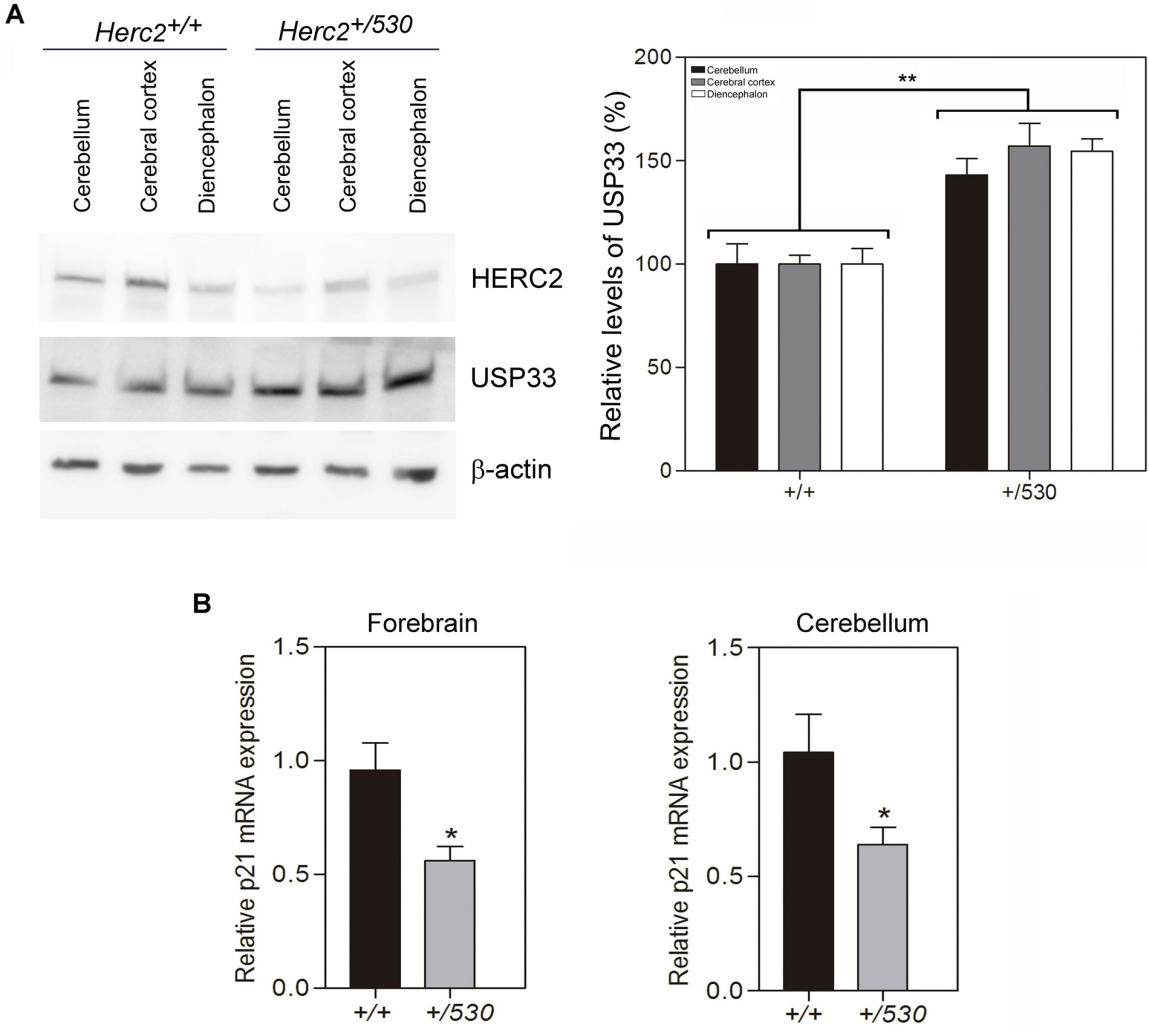
*Herc2^+/530^* mice show reduced activity of HERC2 **A.** USP33, a substrate of ubiquitination of HERC2, was analyzed in lysates (cerebellum, cerebral cortex and diencephalon) from 8 week old mice by immunoblotting. Higher levels of USP33 were observed in all areas of *Herc2^+/530^* mice. Levels of USP33 were quantified and normalized with respect to β-actin levels. **B.** *Herc2^+/530^* mice show reduced levels of *p21* mRNA. RT quantitative PCR analyses were performed in forebrain and cerebellum from *Herc2^+/+^* and *Herc2^+/530^* mice to quantify *p21* gene expression (*n* = 10). The levels of expression were normalized with respect to *GAPDH* gene expression.

These data show that the levels of HERC2 protein are reduced almost 50% in *Herc2^+/530^* mice tissues and suggest that the HERC2 activity must also decrease in the brain of heterozygous animals. Two activities have been associated with HERC2 protein; an E3 ubiquitin ligase activity which regulates protein levels of USP33, BRCA1 or XPA [21, 22, 25], and an activity as a stimulator of the p53 oligomerization that regulates the transcriptional activity of p53 [32]. To analyze the activity of E3 ubiquitin ligase, we performed immunoblotting experiments in the mouse brain areas with antibodies against substrates ubiquitinated by HERC2. Only the USP33 protein was detected by immunoblotting in these mouse samples (Figure 5A). Interestingly, in *Herc2^+/530^* mice, the levels of USP33 were higher than in control mice. To examine the activity stimulating p53 oligomerization and transcriptional activity, we analyzed the levels of *p21* mRNA by RT quantitative PCR analysis. A decrease in *p21* mRNA levels was observed in *Herc2^+/530^* mice (Figure 5B). Altogether, these data show that the partial inactivation of the *Herc2* gene in *Herc2^+/530^* mice is sufficient to reduce HERC2 protein levels and activity.

### A homozygous mutation in human *Herc2* causes an Angelman-like syndrome and reduces the activity of the HERC2 protein

**Figure 6:**
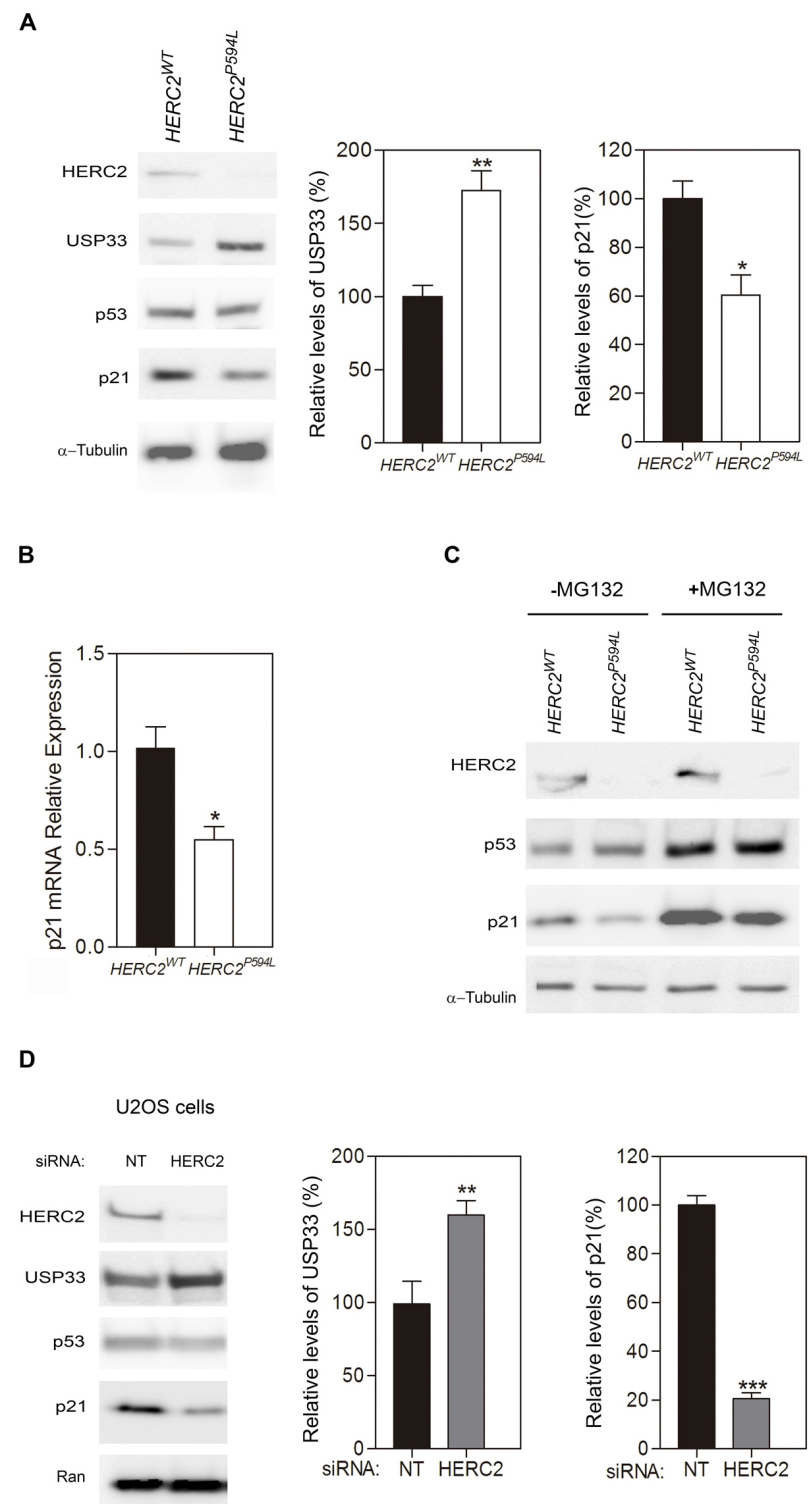
A homozygous mutation in human *HERC2* that causes an Angelman-like syndrome reduces the activity of the HERC2 protein **A.** Fibroblasts derived from individuals with HERC2 wild-type or HERC2 with the mutation P594L were analyzed by immunoblotting for the indicated antibodies. Levels of USP33 and p21 proteins were quantified and normalized with respect to α-tubulin levels. **B.** Levels of *p21* mRNA were analyzed by RT quantitative PCR analysis and normalized with respect to *18S* gene expression. **C.** The levels of HERC2, p53, p21 and α-tubulin proteins were analyzed in the presence or absence of the proteasome inhibitor MG132. **D.** U2OS cells were transfected with non-targeting (NT) or HERC2 siRNAs and analyzed by immunoblotting against the indicated proteins. The levels of USP33 or p21 were quantified and normalized with respect to Ran levels.

HERC2 has been implicated in a human disorder with some features similar to Angelman syndrome. The substitution of proline by leucine at amino acid position 594 in HERC2 caused HERC2^P594L^ instability and almost total loss of the protein in homozygosis [4, 5]. Based on our data from heterozygous animals, we hypothesized that these patients would have lower levels of HERC2 activity. To test this hypothesis, we analyzed the levels of USP33 protein from fibroblasts derived from an affected individual and a healthy control. We observed a high increase of USP33 levels in fibroblasts from a patient (Figure 6A). To confirm that HERC2 activity was diminished, we also analyzed the levels of p21 protein. We observed a decrease in the p21 protein levels in fibroblasts from this patient (Figure 6A). However, the p53 and α-tubulin levels did not significantly change. The *p21* mRNA levels also confirmed the decrease in p53 transcriptional activity in fibroblasts from the patient (Figure 6B). The level of p53 protein is regulated by the proteasome. In the presence of the proteasome inhibitor MG132, we observed a similar stabilization of p53 protein levels in fibroblasts from the patient and the control (Figure 6C). Under these conditions, we also detected a decrease in p21 protein levels in fibroblasts from the patient (Figure 6C), indicating a decrease in its p53 activity. These data show the low activity of HERC2 in individuals carrying the HERC2^P594L^ mutation.

In conclusion, the low HERC2 activity found in *Herc2^+/530^* mice (Figure 5) and in individuals with the HERC2^P594L^ mutation (Figure 6A-6C) correlated with an increase in levels of the USP33 protein and a decrease in levels of the p21 protein. These data suggest that in conditions with low levels of HERC2 protein, cells could contain more USP33 and less p21. To confirm this point, human U2OS cells were depleted of HERC2 using interference RNA, and USP33 and p21 were analyzed by immunoblotting (Figure 6D). HERC2 knockdown increased levels of the USP33 protein and decreased levels of the p21 protein.

### HERC2 regulates motor coordination

**Figure 7:**
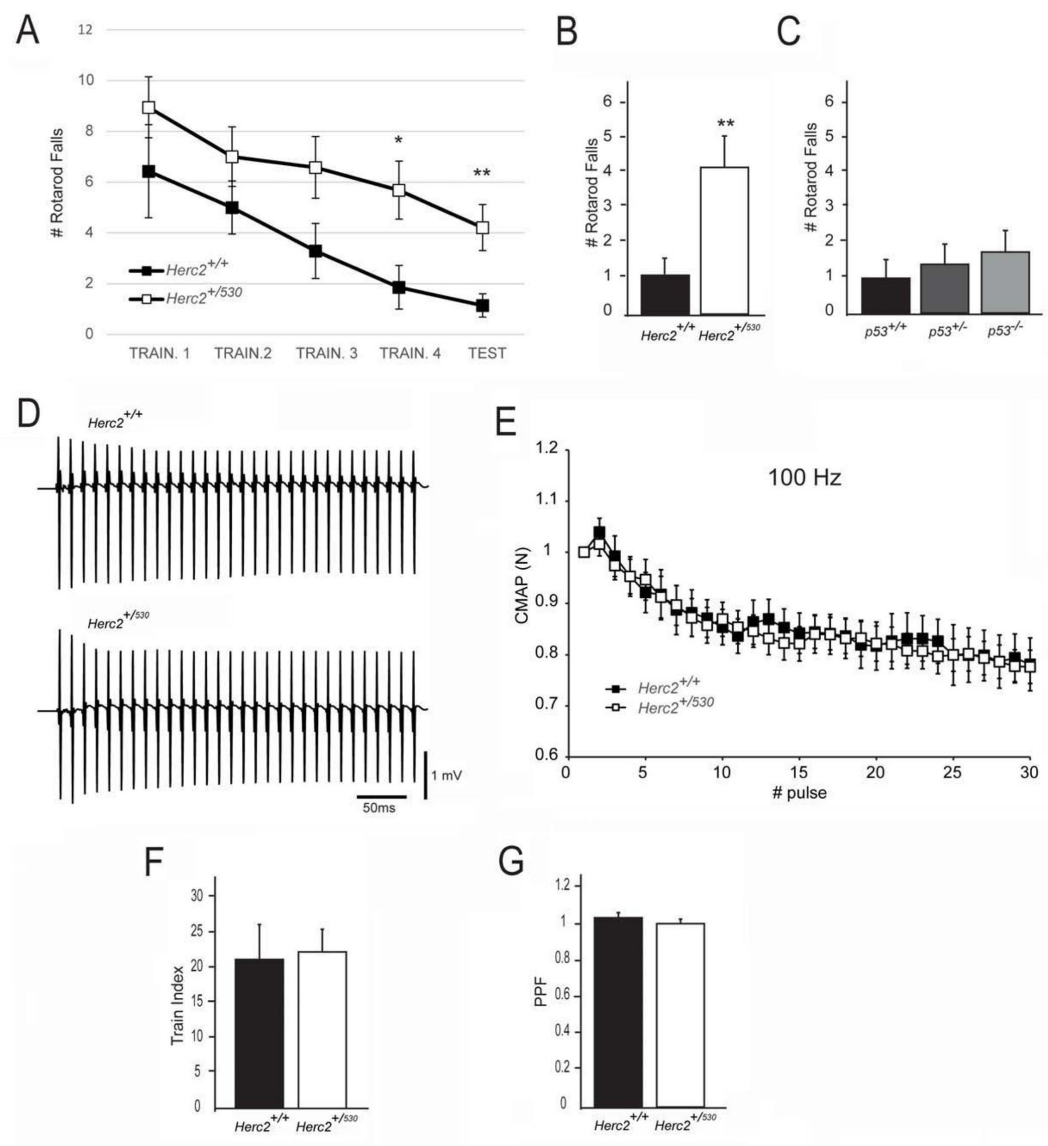
Impaired motor coordination in *Herc2^+/530^* mice **A.**-**B.** The number of falls from the rotarod increases in *Herc2^+/530^* mice at 6 months of age in comparison with control littermates. **C.** No difference was found in *p53^+/−^* and *p53^−/−^* mice in comparison with WT. (D-E) EMG measurements of CMAP amplitudes in the MG of control and *Herc2^+/530^* mice show normal neurotransmission efficacy in postnatal heterozygous mice. **D.** Representative recordings during a train of stimuli at 100 Hz in a control and a *Herc2^+/530^*mouse. **E.** Depression of CMAP amplitudes (normalized to the first response) during a train of stimuli of 300 ms at 100 Hz in control (*n* = 7) and heterozygous mice (*n* = 18). The train index **F.**, corresponding to the depression at the end of the train, and PPF (Pair -Pulse Facilitation) **G.**, are similar between groups.

Individuals carrying the HERC2^P594L^ mutation present a severe developmental delay with an unstable gait [4, 5]. We wondered whether *Herc2^+/530^* mice with a reduction of approximately 50% in HERC2 protein could have some features found in HERC2^P594L^ patients. To examine this question, behavioural tests to measure different cognitive tasks were performed in *Herc2^+/530^* and wild-type mice. Except for the rotarod test, no significant differences between the two groups were found (Table 1; Supplementary Video). *Herc2^+/530^* mice displayed a statistically significant increase in rotarod falls compared to wild-type mice (*Herc2^+/+^*: 1.14 ± 0.46, *n* = 7 and *Herc2^+/530^*: 4.21 ± 0.90, *n* = 19, *p* = 0.0058) (Figure 7A-7B), suggesting a role for HERC2 in motor coordination. Because HERC2 regulates p53 activity, we asked whether p53 inactivation could produce effects similar to *Herc2^+/530^* mice. We performed the rotarod test in *p53^+/−^* and *p53^−/−^* mice. No significant differences were observed compared to wild-type mice (Figure 7C). These data indicate that the impaired motor coordination in *Herc2^+/530^* mice is specific and independent of p53. Next, we analyzed whether muscular function was affected in *Herc2^+/530^* mice. The electrical neuromuscular properties of the *Herc2^+/530^* mice were studied *in vivo* by performing electromyography (EMG) on the medial gastrocnemius (MG) muscle using short train stimuli at 100 Hz (Figure 7D). The depression at the end and the facilitation at the beginning of a train of 30 stimuli were similar in mutant and wild-type mice when measured by normalization of the CMAP (Compound Muscular Action Potentials) amplitude (*p* = 0.8 and *p* = 0.85, respectively) (Figure 7E-7G). These results indicate a normal muscular function.

**Table 1:** Behavioural tests of Herc2^+/530^ mice

Cognitive task	Behavioural tests		Test Values: mean ± error (n)		*P* value
			*Herc2^+/+^*	*Herc2^+/530^*	
Anxiety	Time outside of the dark box (s)		235 ± 13 (9)	232 ± 9 (8)	0.83
	Immobile time suspended by the tail (s)		205 ± 14 (9)	173 ± 14 (19)	0.11
Learning and Memory	Object recognition memory (DI)	STM	0.24 ± 0.08 (6)	0.23 ± 0.05 (10)	0.87
		LTM	0.26 ± 0.09 (6)	0.32 ± 0.06 (10)	0.60
	Step through passive avoidance test (Latency)	STM	2.92 ± 0.24 (6)	2.21 ± 0.37 (15)	0.12
		LTM	2.84 ± 0.31 (7)	3.04 ± 0.32 (19)	0.66
Motor function	Open field (total activity)		1649 ± 108 (8)	1612 ± 199 (16)	0.87
	Rotarod (#Falls)		1.14 ± 0.46 (7)	4.21 ± 0.90 (19)	**0.0058
	Forelimb Grip strength (s)		7.00 ± 0.98 (6)	7.96 ± 0.78 (8)	0.46

STM: Short Term Memory; LTM: Long Term Memory; DI: Discrimination index; n: number of animals

**Figure 8:**
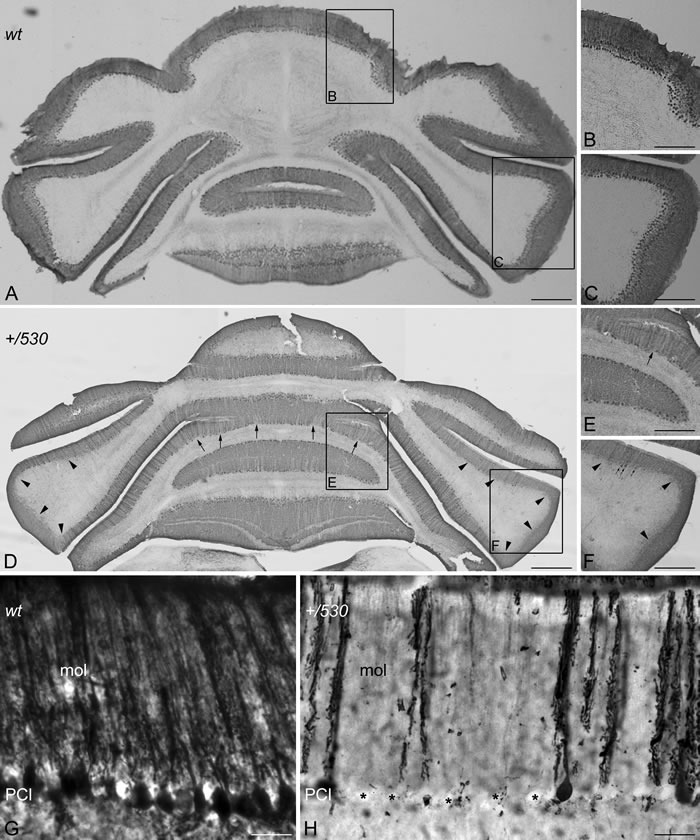
Purkinje cells loss in *Herc2^+/530^* mice Microphotographs of coronal sections through the cerebellar cortex of 9 months old wild-type (*wt*, **A**-**C**, **G**) and 9 months old *Herc2^+/530^* mice (*+/530*, **D**-**F**, **H**). Calbindin immunohistochemistry shows as Purkinje cell somata form a continuous cell layer in the *Herc2^+/+^* (*wt*) cerebellum (A-C) However, parasagittal zones lacking of immunoreactivity throughout the *Herc2^+/530^* cerebellum indicative of Purkinje cell loss are observed (arrows and arrowheads, in (D, F)). These symmetrical Purkinje cells deprived bands, characterized by the presence of wide spaces lacking Purkinje cell somata (H, asterisks) and dendritic debris through the molecular layer (H, mol), distribute differently according a medio-lateral gradient. In the vermis and paravermal zones the immunonegative zones are sagittaly distributed in narrow gaps (D-E, arrows); while at the hemispheres the areas devoid of Purkinje cells, also bilateral, reach a greater extension (D, F, arrowheads) in which remain some surviving Purkinje cells (F, small arrows). G, illustrates the *Herc2^+/+^* (*wt*) immunostaining of normal Purkinje cells; note as dendritic trees fulfill the molecular layer (G, mol), while Purkinje cells somata align in a continuous row. PCl, Purkinje cells layer. Bars = 600 μm (A, D), 400 μm (B-C, E-F), and 30 μm (G-H)

**Figure 9:**
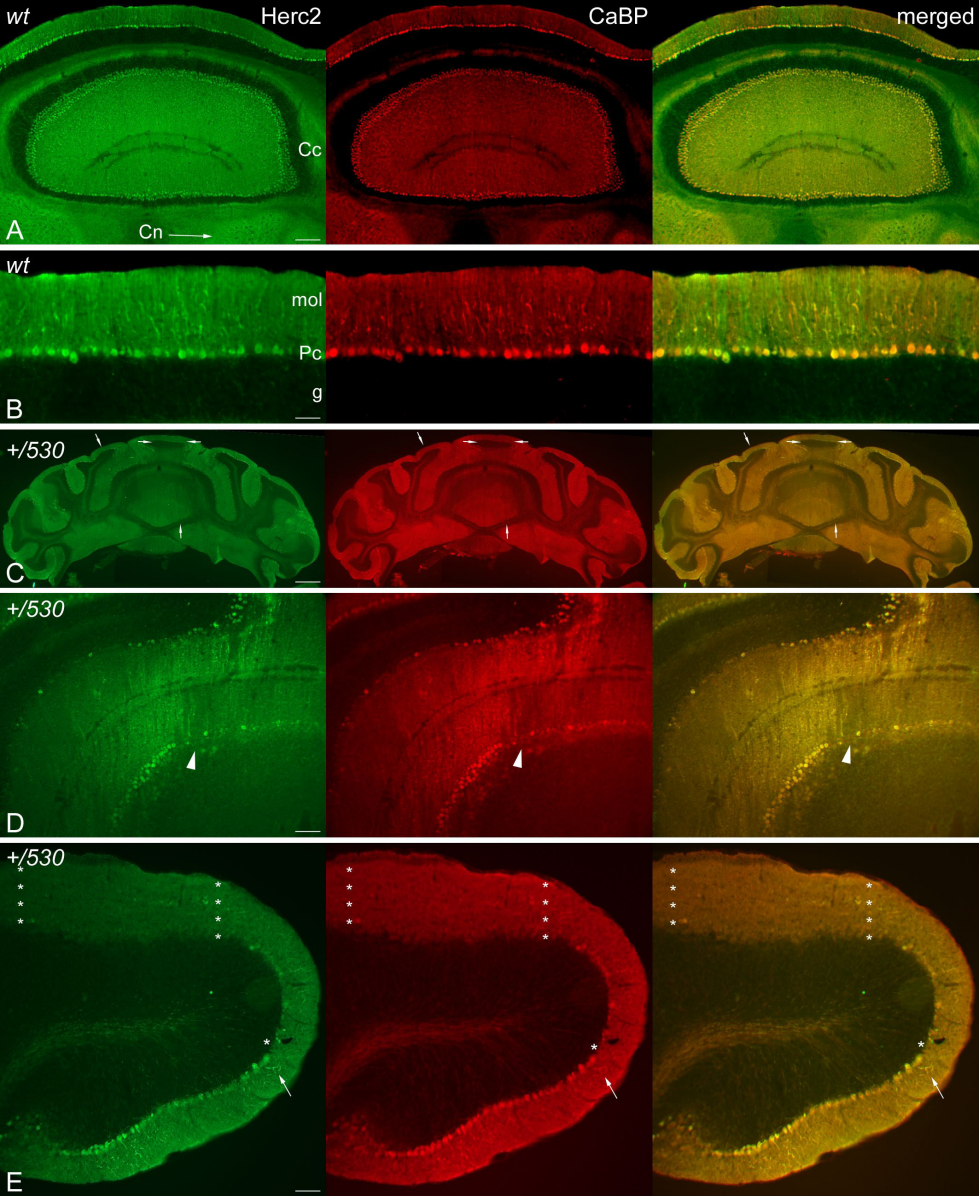
HERC2 is present in Purkinje cells Microphotographs of coronal sections through the cerebellar cortex of 9 months old *Herc2^+/+^* (*wt*, **A.**-**B.**) and 9 months old *Herc2^+/530^* mice (*+/530*, **D.**-**E.**). Epifluorescence microscopy analysis shows that HERC2 is expressed in all the adult Purkinje cells colocalizating with the general marker of Purkinje cell calbindin (CaBP) (A-E) in the cerebellar cortex (A, Cc,), and their axonal endings in the cerebellar nuclei (A, Cn, arrow). *Herc2^+/530^* cerebellum displays parasagittal bands of Purkinje cells loss in the vermis and paravermal zones (**C.**, arrows; D, arrowheads), and areas of extensive Purkinje cell loss in the cerebellar paraflocculus (asterisks). The arrows in E illustrate the co-expression of both proteins in the Purkinje cell dendritic tree. Bars = 750μm (C), 200 μm (A), 100μm (D), 75μm (E), and 50 μm (B).

The cerebellum regulates motor coordination, and alterations in its structure have been associated with impaired motor coordination. To examine whether the deficit of motor synchronization in *Herc2^+/530^* mice was caused by an alteration of cerebellar structure, we performed immunohistochemistry analysis of the specific marker of Purkinje cells calbindin-D28 k (CaBP) (Figure 8). CaBP immunoreactive Purkinje cell somata form a continuous cell layer in the cerebellar cortex of *Herc2^+/+^* mice (Figure 8A-8C). In *Herc2^+/530^* mice, parasagittal zones devoid of immunoreactivity throughout the cerebellum indicative of Purkinje cell loss were observed (arrows and arrowheads in Figure 8D-8F). These symmetrical Purkinje cell-deprived bands -characterized by the presence of wide spaces lacking cell somata in the Purkinje cells layer (asterisks in Figure 8H) and dendritic debris through the molecular layer (mol in Figure 8H)-, were distributed differently along a medio-lateral gradient (see comparison between Figure 8D-8F from a *Herc2^+/530^* mouse and Figure 8A-8C from a *Herc2^+/+^* mouse). Thus, vermal and paravermal Purkinje cells were less affected than in the cerebellar hemispheres. In the vermis and paravermis, the loss of Purkinje cells was distributed in narrow gaps (arrows in Figure 8D and 8E), while at the hemispheres, the areas of Purkinje cells loss reached a great extension (arrowheads in Figure 8D and 8F), in which remain some surviving Purkinje cells (small arrows in Figure 8F). Epifluorescence microscopy analysis showed that HERC2 immunoreactivy in Purkinje cells colocalize with CaBP (Figure 9). In *Herc2^+/530^* mice, HERC2 immunohistochemistry confirmed the loss of Purkinje cells in narrow gaps at the vermis/paravermis or in a greater extension at the hemispheres (Figure 9C-9E).

**Figure 10:**
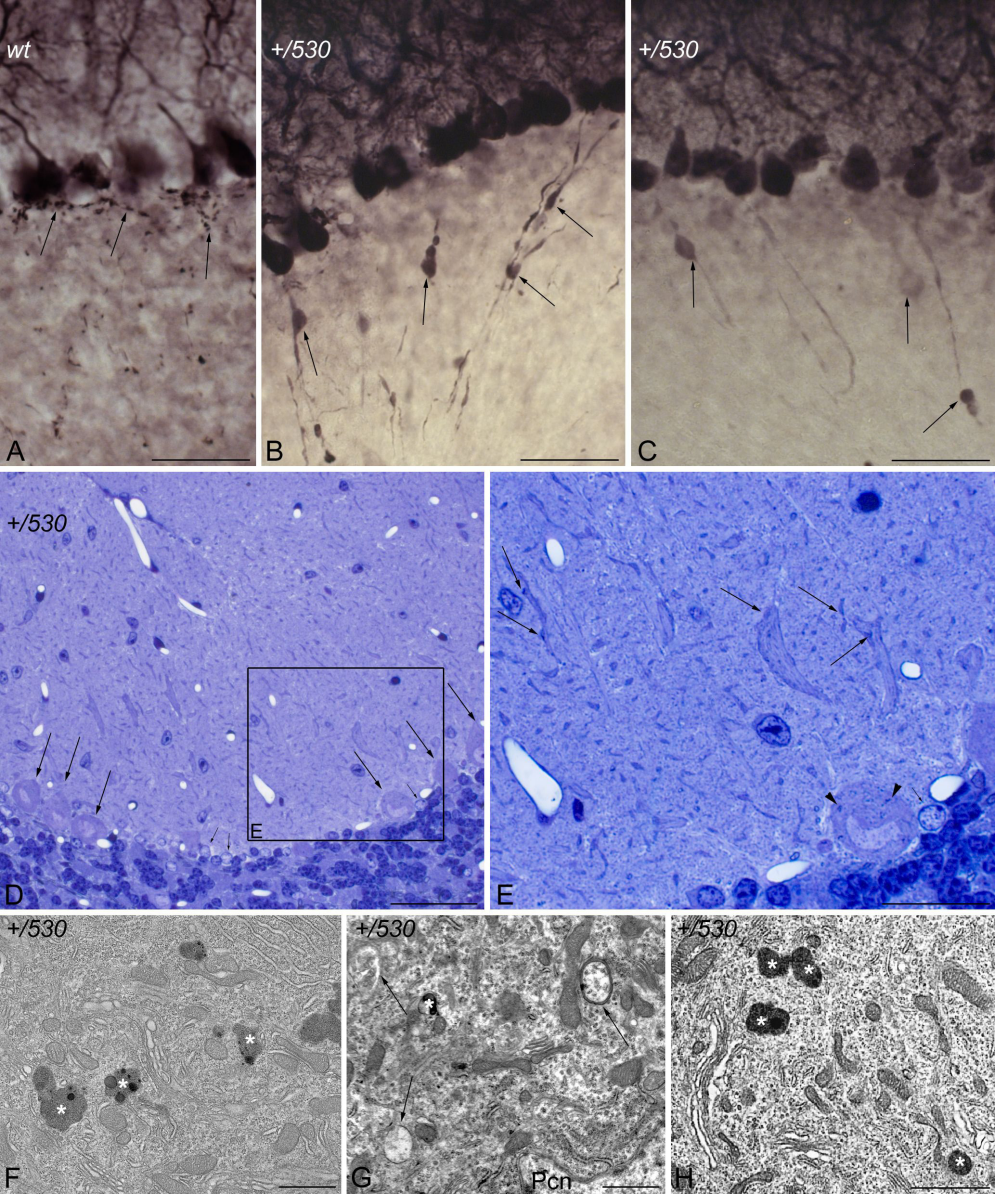
Purkinje cell degeneration in *Herc2^+/530^*mice Microphotographs of transmitted light **A.**-**E.** and electron microscopy **F.**-**H.** of parasagittal sections through the cerebellar cortex of 9 months *Herc2^+/+^* (*wt*, A), and 2 (H) and 9 months old *Herc2^+/530^* mice (*+/530*, **B.**-**G.**). Calbindin immunohistochemistry reveals the presence of rounded thickenings resembling to axonal torpedoes in *Herc2^+/530^* Purkinje cell axons (B-C, arrows), which contrast with the fine grained morphology of normal Purkinje cells axonal plexuses (A, arrows). 1.5 μm thick sections illustrated Purkinje cells (D, arrows) limiting a zone in which disappeared Purkinje cells were substituted by glial Golgi-epithelial cells (small arrows in D and E). High magnification allows detect degenerative dark accumulations within the cytoplasm of the soma (E, arrowhead) and the dendrites (E, arrows) of the Purkinje cells. *Herc2^+/530^* Purkinje cells cytoplasm possesses lysosomes, electron-dense debris (F-H, asterisks), and autophagosomes with different degrees of evolution (G, arrows). Pcn, Purkinje cell nucleus. Bars = 50 μm (A-D), 25 μm (E), and 1 μm (F-H).

**Figure 11:**
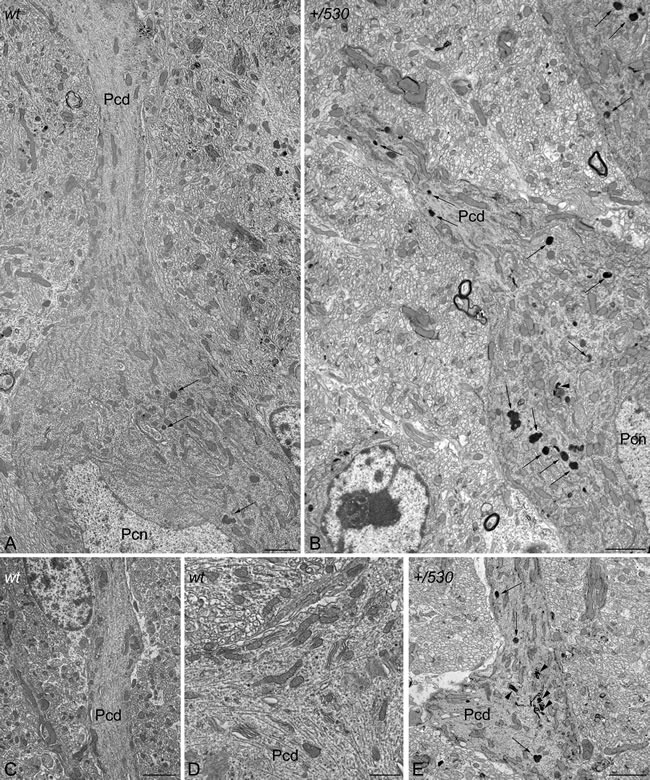
Ultrastructural analysis of *Herc2^+/530^* mice indicates accumulation of autophagosomes and lysosomes in Purkinje cells Electron photomicrographs of parasagittal sections through the cerebellar vermis of 9 months old *Herc2^+/+^* (*wt*, **A.**, **C.**-**D.**) and 9 months old *Herc2^+/530^* mice (*+/530*, **B.**, **E.**). An important difference in the presence of autophagic (arrowheads), and lysosomal (arrows) organelles can be observed between wild-type (A) and *Herc2^+/530^* (B) Purkinje cells cytoplasm. The difference is even most evident in the principal Purkinje cell dendrites. Thus, numerous degenerative signs are present in *Herc2^+/530^* Purkinje cells dendrite (E), while are almost absent in wild-type ones (C-D). Pcd, Purkinje cell dendrite. Pcn, Purkinje cell nucleus. Bars = 2 μm (A-C, E), and 1 μm (D).

**Figure 12:**
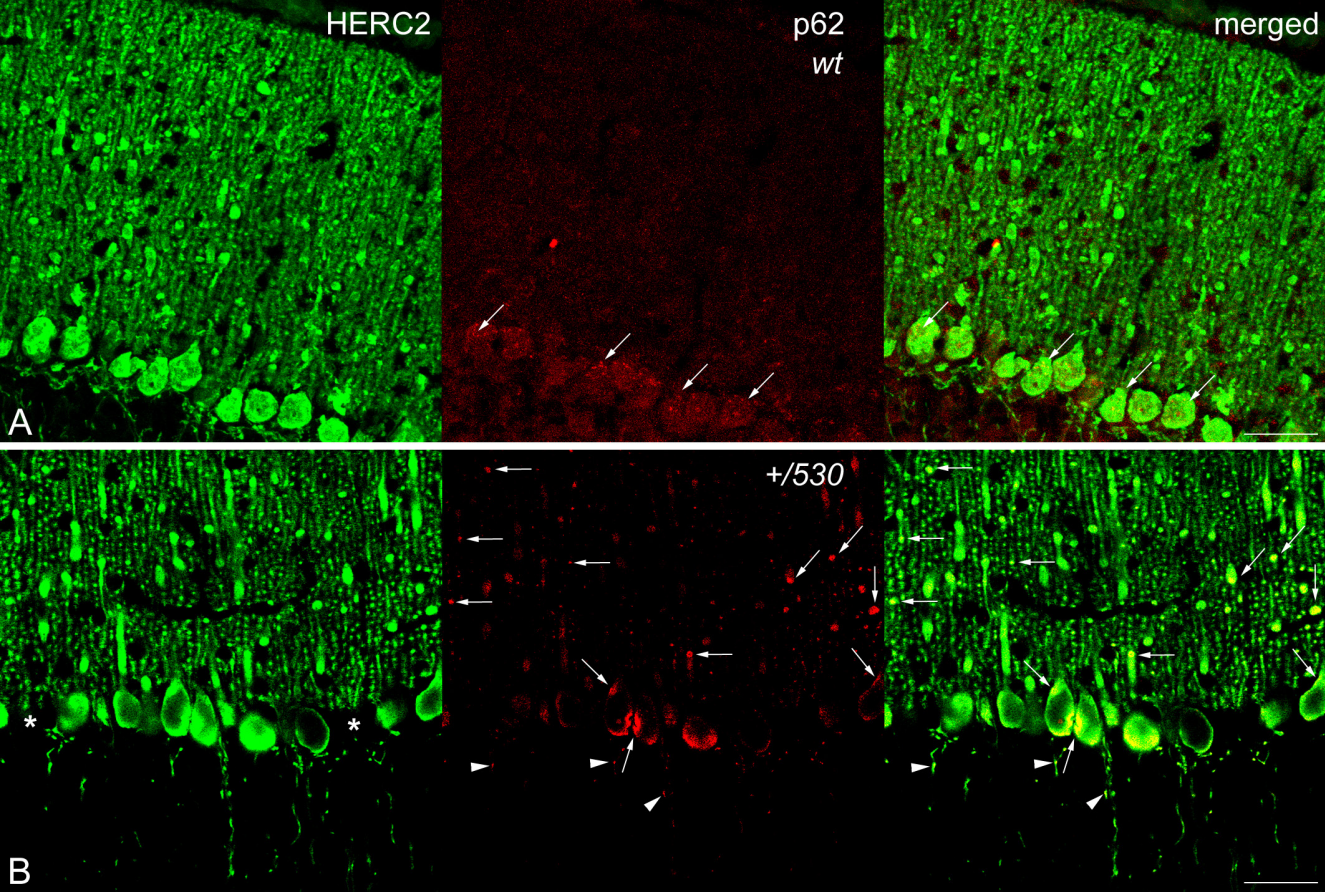
p62/SQSTM1 in *Herc2^+/530^* mice Laser confocal microphotographs of coronal sections through the cerebellar cortex of the vermis of 9 months *Herc2^+/+^* (*wt*, A) and 9 months old *Herc2^+/530^* (*+/530*, B) mice double labeled with HERC2 and p62 antibodies. Colocalizations of HERC2 and p62 are indicated by arrows in dendrites and cell somata **A.**, **B.**, and by arrowheads in the axonal torpedoes of *Herc2^+/530^* Purkinje cells (B). Asterisks in B indicate the absence of Purkinje cell bodies. Bar = 50 μm (A-B).

CaBP immunohistochemistry also revealed the presence of pathological signs in *Herc2^+/530^* Purkinje cells. Rounded thickenings resembling axonal torpedoes were observed in Purkinje cell axons, which contrast with the fine grained morphology of normal Purkinje cells axonal plexuses (see arrows in the comparison between *Herc2^+/+^* and *Herc2^+/530^* in Figure 10A-10C). Phenotypic alterations of *Herc2^+/530^* Purkinje cells were also observed in the 1.5 μm thick sections stained with toluidine blue (Figure 10D-10E). Disappeared Purkinje cells somata were substituted by glial Golgi-epithelial cells (Figure 10D: arrows, Purkinje cells; small arrows, Golgi-epithelial cells). High magnification allowed to detect degenerative dark accumulations within the cytoplasm of the soma (arrowheads in Figure 10E) and the dendrites (arrows in Figure 10E) of the Purkinje cells. Electron microscopy analysis of cerebella confirmed these degenerative signs in *Herc2^+/530^* mice (Figure 10F-10H and 11). The cytoplasm of *Herc2^+/530^* Purkinje cells contained a high number of lysosomes and electron-dense debris (Figure 10F-10H, asterisks), and autophagosomes with different degrees of evolution (Figure 10G, arrows). Damaged cisterns of Golgi apparatus and numerous cisterns of the rough endoplasmic reticulum fused to the cytoplasmic face of the nuclear membrane were also observed (not shown). The difference between mice *Herc2^+/+^* and *Herc2^+/530^* was even most evident in the principal Purkinje cell dendrites (Pcd in Figure 11). Thus, numerous degenerative signs were present in *Herc2^+/530^* Purkinje cells dendrite (Figure 11B and 11E), while were almost absent in wild-type ones (Figure 11A, 11C and 11D). These degenerative signs were found in 2 and 9 month-old animals (Figure 10F-10H), with a slight increase of these alterations in the older mice (not shown). These data show that the partial inactivation of HERC2 in *Herc2^+/530^* mice causes Purkinje cell loss, which explain motor incoordination detected here in rotarod test. The increase of autophagosomes and lysosomes observed in *Herc2^+/530^* Purkinje cells (Figures 10 and 11) led us to wonder whether HERC2 may be involved in the regulation of autophagy. To this end, we have analyzed the adaptor-substrate p62/SQSTM1 by laser confocal microscopy. HERC2 immunostaining colocalized with p62 (Figure 12). This colocalization, it was observed both in wild-type animals as in *Herc2^+/530^* animals (Figure 12, arrows and arrowheads in the merged). *In Herc^+/+^ mice*, p62 immunostaining showed a punctate labeling pattern that concentrated in Purkinje cells somata (Figure 12, arrows). This pattern was altered in *Herc2^+/530^* animals; thus, in addition to the somatic labeling, p62 immunostaining was also evident within the dendritic trees and the axonal torpedoes of Purkinje cells (Figure 12, arrows and arrowheads) indicating a dysregulation of autophagy in *Herc2^+/530^* Purkinje cells. Altogether, these findings suggest that HERC2 plays an active role in regulating the Purkinje cells homeostasis, whose deregulation elicits alterations in motor coordination.

## DISCUSSION

We have generated a new mutant allele of the HERC2 ubiquitin ligase, *Herc2^530^*, which has led us to identify *Herc2* as an essential gene for embryonic development. The *HERC2* gene encodes an unusual long polypeptide chain of almost 5,000 amino acid residues. The maintenance during evolution of this single and long polypeptide chain suggests an important physiological role for the giant HERC2 protein. Here, we showed that the inactivation of *Herc2* causes embryonic lethality before 7.5 days. At that time, we observed a high number of abnormal placentas, probably indicating resorption of unviable embryos. Although we have not studied the cause of this embryonic lethality, a possibility could be defective implantation. The unviability of embryos when HERC2 is inactivated would be indicative of the difficulty of finding human individuals homozygous for mutations of this gene. To our knowledge, only the HERC2^P594L^ mutation found in the Amish community has been reported in humans [4, 5]. In these individuals, the HERC2 protein is unstable and low levels are detected, although apparently high enough to avoid embryonic lethality. Ubiquitin ligases that interact with p53, such as MDM2 or MDM4, had also been identified as essential during embryonic phases. The absence of MDM2 induces embryonic lethality in mice at the peri-implantation (E4-E5.5) stage of development, whereas mice deficient for MDM4 die in mid-gestation (E7.5-E8.5) [33–35]. The lethality during these phases was rescued by a double knockout of the ubiquitin ligase and p53 [33–35]. Because HERC2 is also an ubiquitin ligase that interacts and regulates p53 activity [32], we analyzed whether the double knockout of HERC2 and p53 could also rescue the lethality. Our results show that the double knockout did not rescue the embryonic lethality, indicating a p53-independent role for HERC2 in development. Moreover, these data also confirm, at a genetic level, the different functions of MDM2 (or MDM4) and HERC2 in p53 regulation.

HERC2 acts as an ubiquitin ligase tagging for degradation by the proteasome of substrates such as XPA, BRCA1, NEURL4, FBXL5 or USP33 [21, 22, 24–26]. HERC2 may also positively regulate p53 activity through stimulation of its oligomerization [32]. The analysis of levels of USP33 and p21 led us to analyze these two activities, respectively. These activities had been reported in culture cell but not in physiological conditions. The generation of *Herc2^+/530^* mice containing approximately one-half of the HERC2 protein (Figure 4) led us to analyze the role of HERC2 *in vivo*. *Herc2^+/530^* mice had higher levels of USP33 protein and lower levels of p21 mRNA (Figure 5). These results show for the first time the inhibition of HERC2 activity in physiological conditions. Individuals from the Amish community with a punctual mutation in HERC2, HERC2^P594L^, suffer a developmental disorder with features similar to Angelman syndrome [4, 5]. These individuals produce an instable HERC2^P594L^ protein. Skin fibroblasts obtained from these individuals also had higher levels of USP33 protein and lower levels of p21, in agreement with a loss of HERC2 activity. We showed how these results can be extrapolated to other human cells through interference RNA experiments. HERC2 knockdown caused an increase in the levels of USP33 protein and a decrease in the levels of p21. In conclusion, we show how the loss of HERC2 protein correlates with the loss of its activities, confirming the involvement of HERC2 in cellular processes regulated by USP33 [25] and p53 [32].

The tumor suppressor gene *p53* is mutated in approximately 50% of human sporadic cancers and in inherited cancer predispositions, such as Li-Fraumeni syndrome [27, 28]. Homozygous *p53^−/−^* mice are highly prone to cancer, particularly T-cell lymphoma and sarcoma [36, 37]. Experiments with heterozygous *p53^+/−^* mice indicate that a mere reduction in p53 levels may be sufficient to promote tumorigenesis [38]. The p53 transcriptional activation is dependent of its oligomerization state [29], and p53 mutations that impair its oligomerization have been associated with the Li-Fraumeni syndrome [30, 31]. Because HERC2 interacts with p53 and modulates its transcriptional activity by regulating its oligomerization [32] and *Herc2^+/530^* mice have less p53 activity (Figure 5), we would expect *Herc2^+/530^* mice to show an increased susceptibility to developing spontaneous tumors. However, we have not observed a greater number of tumors in *Herc2^530^* mice (data not shown). In agreement with these data, an increased susceptibility to develop spontaneous tumors in individuals from the Amish community with the HERC2^P594L^ punctual mutation [4, 5] and lower p53 activity (Figure 6) has not been reported. The hypothesis that loss of transcription in p53 is the driving force selected during tumorigenesis needs to be reevaluated, as it is far from straightforward. For example, mice expressing p53 mutants transcriptionally defective for growth arrest, senescence and apoptosis, are not prone to cancer [39, 40]. Moreover, cells from mice deficient for the three p53 target genes, *p21*, *Puma* and *Noxa*, are deficient in their ability to undergo p53-mediated cell-cycle arrest, apoptosis, and senescence, although the animals remain tumor-free [41].

Our study identifies a new function for the HERC2 ubiquitin ligase as a regulator of motor coordination through regulation of Purkinje cells homeostasis. HERC2 protein is expressed in Purkinje cells (Figure 9). The decrease of its protein levels correlates with the loss of Purkinje cells in the vermis and hemispheres of the cerebellum that would explain the motor incoordination detected in *Herc2^+/530^* mice with rotarod experiments (Figures 7 and 8). The Purkinje cell loss is bilateral and symmetrical as in other mutations characterized by the Purkinje cells loss [42], demonstrating the specificity of the degenerative process. This loss is not homogeneously distributed; thus, the loss of Purkinje cells appears as discrete gaps in the continuous staining of the molecular layer of the vermis, while at the hemispheres, the loss reaches a great extension (Figure 8). Despite their homogenous shapes and common morphological features, these results suggest some difference between Purkinje cells and is in agreement with previous observations indicating that these neurons do not constitute a biochemically homogenous population [42, 43]. Pathological signs, such as varicose enlargements resembling axonal torpedoes and widespread accumulations of dense cytoplasmic material, were observed in Purkinje cells of *Herc2^+/530^* mice. Proliferation of Golgi epithelial cells is also indicative of Purkinje cell degeneration. Electron microscopy analysis of *Herc2^+/530^* cerebella showed Purkinje cells containing numerous degenerating signs in autophagosomes, lysosomes and Golgi cisterns (Figures 10 and 11, and data not shown). These results resemble those obtained from a different mutant mouse called *tambaleante* [44, 45]. In these animals, a progressive and specific loss of Purkinje cell was observed with a very similar pattern of neurodegenerative signs. Interestingly, the Purkinje cell degeneration in *tambaleante* mutant mice is a consequence of a missense mutation in HERC1 [44], the structural homolog of HERC2. In this regard, it seems that the functional alteration of one of them it is not compensated by the other. In agreement with this independent role of HERC1 and HERC2, the essential role of HERC1 for normal development and for neurotransmission at the mouse neuromuscular junction [46] was not observed in *Herc2^+/530^* mice (Figure 7). Thus, both ubiquitin ligases seem to have a crucial and independent role in Purkinje cell physiology. This functional similarity of HERC1 and HERC2 was unexpected, because despite their structural homology, endogenous proteins do not interact between them, do not have common known interactors, have different subcellular locations and were involved in different pathways of cell signaling [11, 12, 32]. However, an interaction between the first 1,000 amino acid residues of HERC2 and HERC1 has recently been detected using mass spectrometry analysis [47], suggesting a possible interaction in some unknown circumstances.

A functional role of cerebellar p53 protein in adult walking synchronization has been reported [48]. Because HERC2 positively regulates p53 activity [32] and *Herc2^+/530^* mice had less HERC2 activity, it was plausible that the impaired motor synchronization in *Herc2^+/530^* mice was caused by a decrease in p53 activity. For this reason, we analyzed motor coordination in p53 knockout mice. We did not observe significant differences with rotarod experiments in these animals (Figure 7). Our results are in agreement with other studies where no differences in motor synchronization were detected by Tomasevic *et al*. [49] in p53 knockout mice. These authors described a role for p53 in the recovery of neuromotor function after a traumatic brain injury but not before the injury [49]. Thus, the motor coordination regulated by HERC2 seems to be independent of p53 activity.

Pioneering studies associated mutations at the mouse *Herc2* locus induced by ethylnitrosourea or ionizing radiation with a runty, jerky, sterile phenotype (*rjs*), also known as the juvenile development and fertility phenotype (*jfd2*), characterized by reduced size, jerky gait, fertility problems, including spermatocyte and oocyte abnormalities, defective maternal behaviour, and reduced lifespan with juvenile lethality [50–52]. More recently, a mutation in the *HERC2* gene has been linked to the neurodevelopmental delay and dysfunction seen in Angelman syndrome and autism-spectrum disorders among the Amish community [4, 5]. Now, our study demonstrates an important role of HERC2 in the regulation of motor coordination through Purkinje cells homeostasis that would explain some features observed in *rjs/jdf2* mice and individuals with the HERC2^P594L^ mutation. It is important to note that, different to the above studies, the impaired motor synchronization is observed in mice with only a mutated allele of *Herc2* (*Herc2^+/530^* mice), indicating the relevance of HERC2 activity in motor function coordination.

Studies of spontaneous mouse mutants have implicated autophagy in the death of Purkinje neurons. *Lurcher* mice with mutations in the delta2 glutamate receptor, *pcd* mice with loss of *nna1* expression or *tambaleante* mice with mutation in the HERC1 protein show an increase of autophagy associated with Purkinje cell death [44, 53–55]. While conditional inactivation of autophagy genes such as *Atg5* or *Atg7* from Purkinje cells in mice yields Purkinje cell degeneration and death [56, 57]. This dual role of the autophagy in Purkinje cells degeneration seems to indicate that these neurons are very sensitive to the dysregulation of autophagy. Although more studies will be necessary to establish the role precise of HERC2 in autophagy, our results are in agreement with these previous observations in other mouse models, and reveal a dysregulation of autophagy in *Herc2^+/530^* Purkinje cells. A recent study also suggests the involvement of HERC2 in Parkinson's disease [58], it would be interesting to analyze in future studies the HERC2 role in the midbrain dopaminergic neurons of *Herc2^+/530^* mice.

In summary, the generation of a mutant mouse of *Herc2* led us to identify the HERC2 ubiquitin ligase as essential for embryonic development and an important regulator of motor coordination. These results may also explain some features observed among the Old Order Amish with a homozygous missense mutation in *HERC2*. Future studies will be necessary to identify additional partners involved in HERC2 physiology and its role in human diseases.

## MATERIALS AND METHODS

### Animals

The ES cell line AR0530 from The Sanger Institute containing a gene trap β-galactosidase/neomycin (β-geo-Neo) cassette that has integrated between exons 2 and 3 in the *Herc2* gene was used. We choose this ES cell line to study *Herc2* gene expression by β-galactosidase activity and to avoid the variability of the already existing *Herc2* mutations (deletions, point mutations, DNA rearrangements) [50]. ES cells were injected into 4.5 day C57BL/6J blastocysts, which were then implanted into pseudopregnant females. The resulting 90-95% coat color chimeras were crossed with C57BL/6 mice to generate the heterozygous animals *Herc2/Herc2^530^ (Herc2^+/530^*). *p53* knockout mice B6.129S2-*Trp53*^tm1Tyj^/J from Jackson laboratories [37] were kindly provided by Dr. J. Martin-Caballero. All animal experiments were performed in accordance with guidelines approved by the Ethical Committee for Animal Experimentation of the University of Barcelona.

### Genotyping

Tail DNA or embryo samples were purified using the NucleoSpin Tissue kit (MACHEREY-NAGEL) according to the manufacturer's protocol. PCR amplification was performed using the primers: 530KO1 5′GGCTGCCCAGTCTCGCCTTG3′ and 530KO04 5′CTGTCACCTCTCCGGGAGAAC3′ for the amplification of the *Herc2* wild-type allele; Gal7 5′TTTCCATATGGGGATTGGTG3′ and Gal8 5′TGTCTGTTGTGCCCAGTCAT3′ for the *Herc2* 530 allele; and p53036 5′ACAGCGTGGTGGTACCT3′ and p53037 5′TATACTCAGAGCCGGCCT p53038 5′ CTATCAAGGCATAGCGTTGG for the genotype of p53. The PCR settings were 94°C for 3 minutes, 94°C for 1 minute, annealing at 60°C for 1 minute and elongation at 72°C for 1 minute, for 35 cycles.

### RT-PCR

To assess trap expression, total RNA was isolated from mice tissues or cells using TRIsure reagent (Bioline). Two μg of total RNA was reverse-transcribed using the cDNA Reverse Transcription kit (Applied Biosystems) and random primers. To assess the trap insertion, PCR was carried out with primers: 530KO02 5′CAGGTCTGACCACCCGGAGG3′; 530KO09 5′GGGAGTTCTCGATTTTGTGC3′; and β-geo40/60 5′AGGGTTTTCCCAGTCACGAC3′. The PCR settings were 94°C for 3 minutes, 94°C for 1 minute, annealing at 55°C for 30 seconds and elongation at 72°C for 45 seconds, for 30 cycles. Additionally, cDNA was sequenced using BigDye 3.1 on an ABI 3130XL genetic analyzer (Applied Biosystems). To analyze the expression of *p21*, RT quantitative PCR was carried out using the ABI Prism 7900 HT fast-real Time PCR system and commercially available Taqman assays (Applied Biosystems): *CDKN1A* (mouse p21: Mm04205640_g1; human p21: Hs00355783_m1); *GAPDH* (mouse GAPDH: Mm99999915_g1; human GAPDH: Hs99999905_m1); and *18S (*Hs99999901_s1). The PCR data were captured and analyzed using the Sequence Detector software (SDS version 2.3, Applied Biosystems).

### Histology and immunochemistry

*X-gal* staining of β-galactosidase activity. Mice were anesthetized and perfused via the left ventricle with 2% paraformaldehyde in a phosphate buffer (0.12 M). All tissues were dissected and fixed in the same solution for 3 hours at 4°C, and later were cryopreserved by immersion in 30% sucrose for 48 h at 4°C. Whole tissues were then embedded into an 8% gelatin and 15% sucrose solution, frozen in liquid nitrogen and stored at −80°C. Before staining for β-galactosidase activity, the tissues were sliced (7-10 μm), prepared on polylysinated slides, and permeabilized with PBS-0.3% Triton. After washing in PBS, the slices were incubated in staining solution (2 mM *X-gal*, 4 mM K_4_Fe(CN)_6_, 4 mM K_3_Fe(CN)_6_, 2 mM MgCl_2_ in PBS) overnight while shaking in the dark at 4°C. After washing in PBS, the slices were stained with neutral red and mounted for microscopic observation.

The protocol to study the cerebellar Purkinje cells was reported previously [46]. Briefly, two and nine month old mice were deeply anaesthetised with pentobarbital (80 mg/kg i.p.), and perfused transcardially with 4% paraformaldehyde in 0.12 M phosphate buffer (PB, pH 7.2). After dissection, the brains were post-fixed overnight in the same fixative and transferred to 30% sucrose in PB until they sank. Sagittal and coronal sections of the cerebellum (40 μm thick) were cut on a freezing microtome, and collected in PBS. The sections were incubated overnight with a polyclonal anti-calbindin D-28k antibody (1:10,000). After washing, the sections were incubated for one hour in a biotinylated secondary antibody (1:500) followed by incubation for one hour in the ABC elite kit (1:400). A mixture of 0.3% DAB-0.6% nickel sulfate-0.1% hydrogen peroxide in PBS was used to reveal the immunoreaction. For double labeling analyses the sections were incubated overnight with the following primary antibodies mixtures: polyclonal anti-HERC2 (1:400)/monoclonal anti-calbindin D-28k (1:1000), and polyclonal anti-HERC2 (1:400)/monoclonal anti-p62 (1:100). After washing, the sections were incubated for one hour in a mixture of Alexa Fluor^®^ 488 donkey-anti-rabbit (1:500) and Alexa Fluor^®^ 594 donkey-anti-mouse (1:500). Images were acquired in a Zeiss Axio-Imager M1 microscope. Laser confocal analyses were made on an Olympus FluoView 1000 upright microscope.

### Electron microscopy

Two and nine month old mice were deeply anaesthetized with pentobarbital (80 mg/kg i.p.), and perfused transcardially with a mixture of 1% paraformaldehyde and 1% glutaraldehyde in 0,12 M phosphate buffer (PB, pH 7.2). Thereafter, the brains were dissected out and immersed overnight in the same fixative. Sagittal slices of the cerebella were cut and immersed in 2% OsO_4_ in PB, stained in a block with ethanolic 0.5% uranyl acetate, dehydrated with an increased gradient of ethanol, and embedded in Durcupan (Fluka^®^). Semithin and ultrathin sections were obtained on a Leica EM UC7 ultramicrotome. Semithin sections were stained with 1% toluidine blue. Ultrathin sections were collected in copper grids (150 and 300 mesh) and observed without counterstaining in a Zeiss Libra EM at 80 kV (CITIUS).

### Behavioural tests

#### Anxiety

To evaluate mice anxiety, two different protocols were used [59]. Tail suspension: mice were suspended above the floor by fixing the end of the tail to wire netting and immobility was scored by manual observation during a 5 min test session. Time outside the dark box: mice were placed in a rectangular arena (55×40×40 cm^3^) with a dark box with a door. The time inside/outside the box was scored by manual observation during a 5 min test session.

### Learning and memory

#### Object recognition memory

Mice were tested as described previously [60]. Briefly, mice were placed in a rectangular arena (55×40×40 cm^3^) and two identical objects were placed in the arena during the training phase. Subsequently, the animal's memory of one of the original objects was assessed by comparing the amount of time spent exploring the novel object as compared with that spent exploring the familiar one. The relative exploration of the novel object was expressed as a discrimination index [DI 5 (t_novel_ - t_familiar_)/(t_novel_ + t_familiar_)].

#### Step-through passive avoidance test

The test was performed as described previously [60]. Briefly, in the habituation phase, the mice were handled and allowed to move freely for 1 min in a chamber (47×18×26 cm^3^, manufactured by Ugo Basile). In the training phase, the mice were confined to the light compartment and then 30 s later, the door separating the dark-light compartments was opened. Once mice entered the dark compartment, the door closed automatically and the mice received an electrical stimulation (0.5 mA, 5 s) delivered through the metal floor. In the retention tests, the latency to enter into a dark compartment (escape latency) is a measure of information learning or memory retention. To compare the results obtained in different experiments, the fold change in escape latency with respect to the latency obtained in the training session is calculated.

### Motor function

#### Motor activity in the open field

To evaluate locomotor and exploratory activity, mice were placed for 5 minutes in an open field (38×21×15 cm) (Cybertec S.A.). This apparatus consisted of a walled platform containing infrared emitters and sensors (IR) coupled to an altimeter, and the movement sensor was connected to a computer that recorded the number of times the mouse interrupted the IR beams/min.

#### Fore limb grip strength

To evaluate fore limb strength, mice were held above a horizontal wire and lowered to allow the fore limbs to grip the wire. The ability of the mice to remain attached by the fore limbs was scored during 10 s.

#### Rotarod

To habituate mice to the rotarod (Ugo Basile Biological Research Apparatus), the animals were placed on the roller at a speed of 20 rpm until they could remain on it for one minute without falling off. To assay motor coordination, animals were then tested at a rotational speed of 20 rpm, accelerating to 60 rpm in increments of 5 rpm, and quantifying the number of falls at each increase in speed.

### Electrophysiological analysis of the medial gastrocnemius (MG) muscle in mice

Compound muscular action potentials (CMAPs) were recorded in anesthetized mice (tribromethanol 2 %, 0.15 ml/10 g body weight, i.p.) as described previously [46, 61]. Briefly, the recording needle electrode was placed into the medial part of the MG muscle and the reference electrode was situated at the base of the fifth phalanx. A ground electrode was placed at the base of the tail. Stimulating needle electrodes were placed at the sciatic notch and the head of the fibula. Stimulation protocols of supramaximal current pulses (0.05 ms duration, 5-10 mA amplitude) were applied as a short train of 100 Hz pulses generated by an isolated pulse stimulator (Pulse Train Stimulator Cibertec cs-20). The outputs recorded were differentially amplified (P511 AC Amplifier Astro-Med, INC), digitally acquired at 10,000 samples/s (CED 1401 Plus; Cambridge Electronic Designed, Cambridge, UK) and stored on a computer for later analysis. The analysis consisted of measuring the amplitude from the positive to the negative peak of the CMAPs recorded during a train of stimuli, normalizing the amplitude to the first response.

### Cell culture and transfection

Human fibroblasts and ethical statements were previously described [5]. U2OS cells were obtained from ATCC. Cells were cultured at 37°C in Dulbecco's modified Eagle's medium (DMEM) supplemented with 10% fetal bovine serum, 100 units/ml penicillin, 100 μg/ml streptomycin and 2 mM glutamine. Transfection of cells with siRNAs (Non Targeting, NT: UAGCGACUAAACACAUCAA; HERC2, H2: GACUGUAGCCAGAUUGAAA) purchased from GenePharma was carried out using calcium phosphate. Transfected cells were analyzed 72 hours post-transfection. MG132 (Z-Leu-Leu-Leu-al) (Sigma-Aldrich) was added to the cells for 6 hours to a final concentration of 10 μM.

### Antibodies used

The following antibodies were used: anti-HERC2 monoclonal (BD Biosciences); anti-HERC2 polyclonal [32]; anti-p21 (C-19); anti-p62 (SQSTM1 (D-3): sc-28359); anti β-actin (Santa Cruz Biotechnology, Inc.); anti-calbindin D-28k polyclonal (Cb-38a, Swant); anti-calbindin D-28k monoclonal (Cb-955, Sigma); anti-p53 Ab-5 (DO-7) (Neo Markers); anti-USP33 (Proteintech); anti-Ran [62]; anti α-tubulin (Ab-1, Calbiochem); Alexa Fluor^®^ 488 donkey-anti-rabbit (A21207), and Alexa Fluor^®^ 594 donkey-anti-mouse (A21203) (Invitrogen); horseradish peroxidase-conjugated secondary antibodies (Invitrogen); biotin-conjugated secondary antibodies (Vector); and the Avidine-Streptavidine Elite Kit (Vector).

### Lysate and immunoblot

Mice were euthanized by cervical dislocation. The organs were collected and frozen in liquid nitrogen and stored at −80°C until analysis. Tissues, human fibroblasts or U2OS cells were prepared in lysis buffer (consisting of 50 mM TrisHCl, pH 7.5, 150 mM NaCl, 0.5% NP40, 50 mM β-glycerophosphate, 50 mM NaF, 1 mM sodium vanadate, 1 mM phenyl-methylsulfonyl fluoride, 5 μg/mL leupeptin, 5 μg/mL aprotinin, 1 μg/mL pepstatin-A and benzamidine 100 μg/mL) and the tissues were homogenized in a motor-driven Polytron PT3000. The lysates were incubated on ice for 20 minutes and centrifuged at 13,000 g for 10 minutes at 4°C. Total protein levels were measured by BCA (Pierce). Equal amounts of supernatant proteins were analyzed using the Tris-acetate PAGE system [63]. Band intensities were analyzed using a gel documentation system (LAS-3000, Fujifilm). Proteins levels were normalized and expressed as a percentage of controls.

### Statistical analysis

The results are expressed as mean±SEM. The data were analyzed by one-way analysis of variance (ANOVA) or Student's *t*-test. For comparison of significance, Tukey's test was used as a post hoc test according to the statistical program GraphPad Prism. Differences were considered significant at *p* values of less than 0.05: *p < 0.05, **p < 0.01, and ***p < 0.001.

## SUPPLEMENTARY MATERIALS VIDEO


